# Surgical revascularization versus amputation for peripheral vascular disease in dialysis patients: a cohort study

**DOI:** 10.1186/1471-2369-6-3

**Published:** 2005-03-21

**Authors:** Christine M Logar, Lisa M Pappas, Nirupama Ramkumar, Srinivasan Beddhu

**Affiliations:** 1 Renal Section, Salt Lake VA Healthcare System, University of Utah School of Medicine, Salt Lake City, UT, USA; 2 Division of Nephrology & Hypertension, University of Utah School of Medicine, Salt Lake City, UT, USA

## Abstract

**Background:**

Surgical treatment of peripheral vascular disease (PVD) in dialysis patients is controversial.

**Methods:**

We examined the post-operative morbidity and mortality of surgical revascularization or amputation for PVD in a retrospective analysis of United States Renal Data System. Propensity scores for undergoing amputation were derived from a multivariable logistic regression model of amputation.

**Results:**

Of the Medicare patients initiated on dialysis from Jan 1, 1995 to Dec 31, 1999, patients underwent surgical revascularization (n = 1,896) or amputation (n = 2,046) in the first 6 months following initiation of dialysis were studied. In the logistic regression model, compared to claudication, presence of gangrene had a strong association with amputation [odds ratio (OR) 19.0, 95% CI (confidence interval) 13.86–25.95]. The odds of dying within 30 days and within1 year were higher (30 day OR: 1.85, 95% CI: 1.45–2.36; 1 yr OR: 1.46, 95% CI: 1.25–1.71) in the amputation group in logistic regression model adjusted for propensity scores and other baseline factors. Amputation was associated with increased odds of death in patients with low likelihood of amputation (< 33^rd ^percentile of propensity score) and moderate likelihood of amputation (33^rd ^to 66^th ^percentile) but not in high likelihood group (>66^th ^percentile). The number of hospital days in the amputation and revascularization groups was not different.

**Conclusion:**

Amputation might be associated with higher mortality in dialysis patients. Where feasible, revascularization might be preferable over amputation in dialysis patients.

## Background

Nineteen percent of incident dialysis patients have peripheral vascular disease (PVD) [1]. Amputation rate among diabetic dialysis patients is ten times that of diabetic population at large [2]. Over 2-years of follow-up, 2.9% of dialysis patients underwent amputation [3] and in a surgical series, 23% of patients who underwent amputation were on dialysis [4]. Thus, peripheral vascular disease is common and carries considerable morbidity and mortality in dialysis patients. However, the surgical management of PVD in the dialysis population remains controversial. It has been suggested by some authors that because of delayed wound healing and prolonged hospitalization, primary amputation is preferred over revascularization for PVD in dialysis patients while others have argued that a careful selection of dialysis patients for revascularization might result in acceptable outcomes [5-12]. In this study, we examined the post-operative morbidity and mortality of surgical revascularization or amputation for PVD in a large cohort of Medicare dialysis patients.

## Methods

The study design was a retrospective analysis of Medicare patients in the United States Renal Data System (USRDS).

### Study population

Of the Medicare patients initiated on dialysis from Jan 1, 1995 to Dec 31, 1999, those who underwent surgical revascularization or amputation within 6 months of initiation of dialysis were studied. Because information on functional status (shown to be an important predictor of post-surgical outcomes in PVD in dialysis patients) [6] and nutritional status (serum albumin and BMI) were only available at initiation of dialysis, only patients who underwent the surgical procedures in the first 6 months of dialysis were included. The study included only those amputated patients with known PVD, as amputations for diabetic foot are also performed for non-ischemic ulcers. Patients with duplicate entries, previous renal replacement therapies, age < 18 years of age and incomplete follow-up information were excluded. In addition, patients with missing data on serum albumin, height and weight were excluded.

### Data assembly

The Medical Evidence (Form 2728) form is the mandatory form gathered by the Centers for Medicare and Medicaid on patients initiated on chronic maintenance dialysis in the US. The Form 2728 data on demographics (age, gender and race), cause of ESRD (diabetes or others), insurance status (Medicare or non-Medicare), comorbid conditions (coronary artery disease, cerebrovascular disease, peripheral vascular disease, congestive heart failure, malignancy, acquired immunodeficiency syndrome, chronic lung disease), smoking, serum albumin, height, weight and functional ability were used in this analysis [13]. Body mass index (kg/m^2^) was calculated from height and weight.

### Definitions of peripheral vascular disease and procedures

A patient with reported PVD in the Medical Evidence form or ICD-9-CM codes 440.20–440.24, 440.29, 440.30–440.32, 443.81, 443.9 or 785.4 from initiation of dialysis to the first hospitalization for amputation or surgical revascularization was considered to have PVD. The clinical indication for the surgical intervention was identified from ICD-9-CM codes as intermittent claudication (440.21), resting pain (440.22), ulcer (440.23, 707.10–707.15, 707.19), gangrene (440.24 or 785.4) or other/ unknown (not any of the above ICD-9-CM codes). The first procedure (amputation or surgical revascularization) that occurred after initiation of dialysis was identified from Medicare billing data. Amputations were defined as ankle or below amputations (procedure codes 84.12 – 84.15), knee or below amputations (procedure codes 84.15 – 84.16) and above knee amputations (procedure codes 84.17 – 84.19). Amputations of toes (84.11) were not included. Proximal bypass procedures involving the aorta-iliac-femoral-popliteal vessels were identified using procedure code 39.29. Distal bypass procedures were identified using procedure code 39.25, "other peripheral vascular bypass surgery, other than aorta-iliac-femoral bypass to the tibial, peroneal or dorsal artery." This code also designates vascular bypass procedures in the upper limb. However, in an earlier study, only three did not have a distal lower-limb bypass procedure in over 300 hospital discharge records with the procedure code 39.25 [14]. Data on PVD-related procedures before initiation of dialysis were unavailable in the USRDS and were not included in the present study.

### Outcomes

Follow-up duration, mortality and transplantation data were obtained from the United States Renal Data System treatment history, claims and patients files. Patients were tracked until loss to follow-up, transplantation, death, or the administrative censor date of December 31, 1999. Cardiovascular death was defined as cause of death coded in the USRDS death notification form as myocardial infarction, atherosclerotic heart disease, cardiomyopathy, arrhythmia, valvular heart disease, cerebrovascular disease and mesenteric infarction. The number of hospital days (including the index hospitalization) in the first 6 months after the procedure and 30-day and 1-year post-operative mortality were the outcomes of interest.

### Statistical analysis

Baseline characteristics of amputation and revascularization groups were not statistically examined because of the large sample size. The propensity score method was used to account for the confounding that arises because patients whom underwent amputation were not otherwise equivalent to patients in whom surgical revascularization was performed. Stepwise logistic regression model was employed to select patient characteristics independently associated with amputation. Variables examined in the logistic regression model were demographics (age, sex, race), dialysis modality, comorbid conditions (diabetes, coronary artery disease, congestive heart failure, cerebrovascular disease and malignancy), clinical indications (intermittent claudication, resting pain, ulcer, gangrene or other/unknown), hematocrit, nutritional status (BMI and serum albumin) and functional status (required assistance to transfer or ambulate). On the basis of the value of each independent factor multiplied by its beta coefficient, subjects were ranked with respect to their predicted probability (propensity score) of undergoing amputation versus revascularization [15,16].

Thirty-day and one-year mortality in amputation and revascularization groups were examined in a multivariable logistic regression model adjusted for propensity scores, demographics (age, sex, race), dialysis modality, comorbid conditions (diabetes, coronary artery disease, congestive heart failure, cerebrovascular disease and malignancy), clinical indications (intermittent claudication, resting pain, ulcer, gangrene or other/unknown), hematocrit, nutritional status (BMI and serum albumin) and functional status (requires assistance to transfer or ambulate). Similarly, we also examined thirty-day and one-year cardiovascular mortality in amputation versus revascularization groups in a multivariable logistic regression model.

Differences in hospital days in the first six months post-procedure were compared between revascularization and amputation groups by analysis of covariance adjusted for variables described in the above mortality analysis. In addition, the duration of follow-up was used as a covariate to account for shorter duration of follow-up in those who died. Hospital days were log transformed to meet normality assumptions.

### Sensitivity analyses

This study relied on ICD-9 CM codes for indications (intermittent claudication, rest pain, ulcer, gangrene or other/unknown) for amputation. As presence of gangrene is a strong indication for amputation and if ICD-9 CM codes reliably identified those patients with gangrene, a logistic regression model that included clinical indications would better predict amputations than another model that did not include clinical indications. This was tested by examining the c statistic of receiver-operator characteristic (ROC) curves of logistic regression models that included and excluded clinical indications.

It could also be argued that patients who underwent amputation were treated only at advanced stage (gangrene) of peripheral vascular disease and if amputations were performed at earlier stages, the outcomes post-amputation would be superior. If advanced peripheral vascular disease (gangrene) was associated with higher likelihood of undergoing amputation, patients with lower likelihood of undergoing amputation (higher likelihood for undergoing revascularization) would have had less advanced peripheral vascular disease. Thus, examination of amputation versus revascularization outcomes in patients at low, moderate and high likelihood of undergoing amputation might reveal better outcomes post-amputation in the low likelihood group and worse outcomes in the high likelihood group. These three likelihood groups were created using the 33^rd ^and 66^th ^percentiles of propensity scores as cut-points.

As procedure code 39.25 might have included upper limb revascularization procedures, analyses were repeated with including only procedure code 39.29 (proximal bypass procedures involving the aorta-iliac-femoral-popliteal vessels).

## Results

Of the 317,533 patients initiated on dialysis from January 1, 1995 to December 31, 1995, there were 5,916 patients who underwent amputation or revascularization in first six months of initiating dialysis. Patients with missing data on demographics or comorbidity (n = 436) and nutritional status (serum albumin or BMI) (n = 1,153) were excluded. There were 279 patients who underwent amputation and revascularization procedures in the same admission. Some of these patients might have undergone revascularization first followed by an amputation in the same limb and others might have undergone amputation in one limb and revascularization in the other limb. Since ICD-9-CM codes do not provide information on which side the procedure was performed, we could not identify the sequence of events and therefore, excluded these patients. Further, amputations are also performed for indications other than peripheral vascular disease and we excluded 106 amputation patients in whom a diagnosis of peripheral vascular disease could not be established based on Medical Evidence forms or ICD-9-CM codes. Thus, 3,942 patients who underwent amputation (n = 2,046) or revasularization (n = 1,896) were studied.

The mean age of the entire cohort was 70 ± 9 years, 64.2% had diabetes and 92.6% were on hemodialysis. There were 493 (12.5%) deaths within 30 days of the procedure, of which 390 (79.1%) occurred in patients with gangrene. 1,950 patients (49.5%) died in the first year following the surgical procedure.

**Figure 1:**
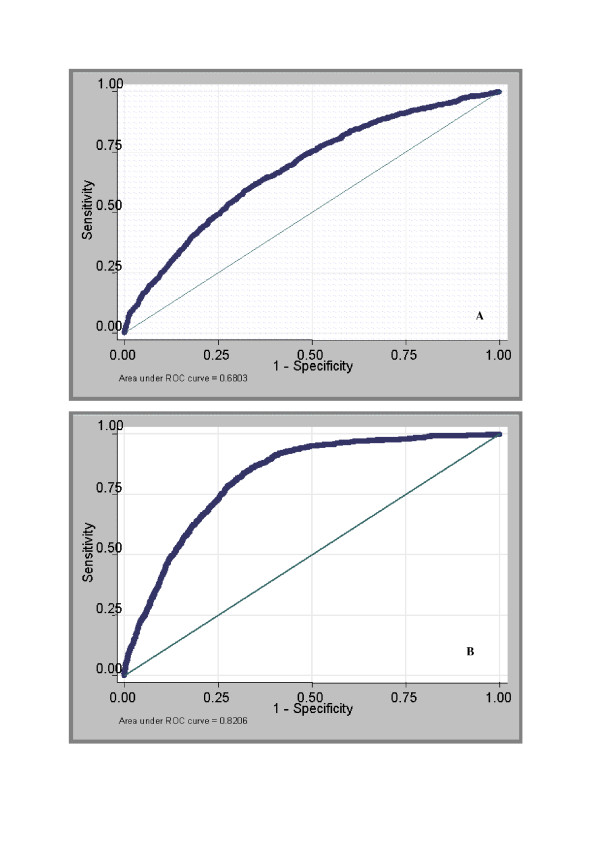
**Receiver-operator characteristic curve of amputation**. Legend: ROC curves of amputation without clinical indication in the model (Figure 1A) and with clinical indication in the model (Figure 1B)

Table 1 summarizes the baseline clinical characteristics in amputation or revascularization groups. Surprisingly, amputation group was younger than the revascularization group. Compared to revascularization group, amputated group had greater prevalence of diabetes (74% v 53%), congestive heart failure (53% v 49%), cerebrovascular disease (19% v 15%) and required assistance to transfer (7% v 2%) or ambulate (17% v 6%). In multivariable logistic regression model (Table 2), presence of gangrene had the strongest association with amputation. Indeed, the area under the ROC curve (Fig 1a) for the logistic regression model that included all variables except clinical indications was only 0.68 indicating low discrimination of these variables in correctly identifying patients whom underwent amputation. When clinical indication was included in the model, the area under the ROC curve (Fig 1b) improved to 0.82 indicating excellent discrimination in identifying patients whom underwent amputation.

There were 165 (8.7%) deaths within 30 days and 942 (49.7%) deaths within 1 year in the revascularization group compared to 328 (16.0%) deaths within 30 days and 1,275 (62.3%) within 1 year in the amputation group. In unadjusted logistic regression analysis, amputation group had 2 fold higher odds of dying within 30 days of the procedure compared to the revascularization group (Table 3). When adjusted for propensity scores and clinical variables, amputated group still had 85% higher odds of dying within 30 days (Table 3). In subgroup analyses by likelihood of undergoing amputation, amputation procedure was associated with the highest odds of dying within 30 days in patients with low likelihood of undergoing amputation, reduced but still significant odds in patients with moderate likelihood of undergoing amputation and non-significant increase in patients with high likelihood of undergoing amputation (Table 3). Similar results were seen when post-operative mortality was examined using follow-up of 1 year. After adjusting for propensity scores, demographics, dialysis modality, comorbid conditions, clinical indications, hematocrit, nutritional and functional status, the amputation group had 46% higher odds of cardiovascular death within 30 days (OR: 1.46, 95% CI: 1.25–1.71) and 18% higher odds of cardiovascular death within 1 year (OR: 1.18, 95% CI: 1.00–1.39).

Clinical indication for the procedure was a predictor of post-operative mortality. There were 6 (6.2%), 12 (10.9%), 16 (6.3%), 390 (15.0%) and 69 (7.9%) deaths associated with intermittent claudication, rest pain, ulcer, gangrene and other/unknown indications respectively. In the above multivariable logistic regression model of mortality in the entire cohort, compared to intermittent claudication, presence of gangrene was associated with 54% higher odds (OR 1.54, 95% CI 1.18 – 2.01, p = 0.002) of death. Presence of rest pain, ulcer or other/unknown indications were not associated with increased risk of death in the multivariable model.

The median number of days spent in the hospital were 28 in the amputation group and 26 in the revascularization group. In unadjusted analysis, these differences were significant (p = 0.003, adjusted R^2 ^= 0.06 for the model). However, these differences were non-significant (p = 0.33, adjusted R^2 ^= 0.08 for the model) when adjusted for propensity scores, demographics (age, sex, race), dialysis modality, comorbid conditions (diabetes, coronary artery disease, congestive heart failure, cerebrovascular disease and malignancy), clinical indications (intermittent claudication, resting pain, ulcer, gangrene or other/unknown), hematocrit, nutritional status (BMI and serum albumin) and functional status (requires assistance to transfer or ambulate).

Among the revascularization group, 1,701 had ICD-9 CM code 39.29, 207 had ICD-9 CM code 39.25 and 12 patients had both codes. When analyses were repeated with revascularization including only ICD-9 CM code 39.29 (proximal bypass procedures involving the aorta-iliac-femoral-popliteal vessels), the results were similar (data not shown).

## Discussion

About 19% of incident dialysis patients have a clinical diagnosis of peripheral vascular disease (PVD)[1]. In the USRDS Dialysis Morbidity and Mortality Study Waves III and IV, 6.1% of diabetic hemodialysis patients and 0.8% of non-diabetic hemodialysis patients underwent lower extremity amputation [3] over two years. When compared to general population, outcomes after revascularization or amputation for PVD in dialysis patients were inferior [8,17]. The problems with revascularization in PVD in dialysis patients include high perioperative and 1-year mortality, decreased wound healing, loss of limb despite patent graft and prolonged hospital stay and poor rehabilitation. Therefore, liberal use of primary amputation for peripheral vascular disease was suggested in dialysis patients by some authors [5]. However, careful patient selection for revascularization in dialysis patients might result in acceptable outcomes. Baele et al [6] reported in a series of 44 hemodialysis patients who underwent revascularization a two-year survival rate of 48%, perioperative mortality of 9%, primary graft patency at 1 year and 2 years of 71% and 63% and limb salvage at 1 year and 2 years of 70% and 52%. In that study, an aggressive approach to limb salvage was favored when patients were found to be ambulatory or able to use the affected extremity for purposes of weight bearing or transfer. Attempted limb salvage was not advocated for patients who were chronically bedridden or those with uncontrolled infection or tissue necrosis precluding a reasonable expectation of limb salvage. Thus, extensive tissue necrosis in non-weight bearing limb and pre-operative infection might be indications for primary amputation.

The results of the current study (Table 2) show that while black race, peritoneal dialysis, diabetes and poor functional and nutritional status were associated with amputation, the strong association of presence of gangrene and younger age with amputation are the most striking. As presence of gangrene was a strong indication for amputation rather than revascularization, either by patient preference or surgical determination of high peri-operative mortality risk, older dialysis patients with gangrene might have never started dialysis or withdrawn from dialysis rather than undergo amputation. Thus, amputation might have been performed only in younger patients with gangrene and not in older patients with gangrene. This might explain the association of younger age with amputation.

As shown in Figures 1a and 1b, presence of gangrene was the overwhelming factor associated with amputation. In an earlier analysis of prevalent hemodialysis patients in the Dialysis Morbidity and Mortality Study Waves 3 and 4 data, elevated serum phosphorus was also found to be a predictor of amputation [3]. Because data on serum phosphorus were unavailable in the Medical Evidence form, we could not examine the association of serum phosphorus with amputation in this study. However, a c statistic of 0.82 (Fig 1b) indicates that the factors considered in this study (Table 2) explained most of the variations in the clinical decision of amputation versus revascularization.

There were 8.7% deaths within 30 days in the revascularization group compared to 16.0% deaths in the amputation group. These results are comparable to earlier reports on peri-operative mortality of dialysis patients who underwent amputation or revascularization [10,12,18,19]. In unadjusted logistic regression analysis, amputation group had 2 fold higher odds of dying within 30 days of the procedure compared to the revascularization group (Table 3). There are several possible explanations for this observation. First, selection of healthier patients with better nutrition and functional status and without significant tissue damage probably resulted in lower 30-day mortality in the revascularized group. However, adjusting for the above factors did not completely eliminate this association. Second, it might be argued that the clinical indications for amputation versus revascularization could explain the differences in outcomes. However, even after using propensity scores to control for confounding by indication, amputation group still had higher mortality. In this retrospective observational study of Medicare data, unmeasured factors that influenced the decision to perform amputation might invalidate the propensity score approach. However, as noted above, a c statistic of 0.82 indicates that the factors considered in this study explained most of the variations associated with the decision to perform amputation. Thus, it is unlikely that other very significant factors that influenced the decision to perform amputation were not considered in this study. Third, it is possible that amputation was only performed in advanced stages of peripheral vascular disease and amputation in earlier stages might have resulted in superior outcomes. If this were true, as explained in the methods section, amputation would be expected to have better outcomes in patients at low likelihood for amputation. This was not the case as shown in Table 3. In fact, amputation in patients with low and moderate likelihood for amputation was associated increased mortality and not in the high likelihood group. These results suggest that undergoing amputation rather than revascularization in healthier dialysis patients with lesser tissue damage might actually increase mortality. Thus, our results support the approach suggested by Baele et al that revascularization is feasible and might be the preferred method of treatment of symptomatic PVD in carefully selected dialysis patients. Therefore, symptomatic peripheral vascular disease in dialysis patients should not automatically result in amputation.

One of the arguments in favor of amputation which results in loss of limb over revascularization that might save the limb is that revascularization is associated with prolonged hospitalization whereas amputation results in shorter hospital stay, quicker recovery and better rehabilitation. However, in this large study, the median (interquartile range) number of hospital days in amputation and revascularization groups were 28 (6–48) and 26 (14–44) days respectively. Thus, these data do not support the contention that amputation is associated with lower hospital stay. Further, in many studies only about 25% patients post-amputation were ambulatory [4,20]. Thus, it does not appear that amputation would result in shorter hospital stay, quicker recovery and better rehabilitation.

A striking feature of the current study is the association of both revascularization and amputation with very high 30-day post-operative mortality in dialysis patients. These results emphasize the importance of preventing or slowing down the development of advanced PVD that requires surgical interventions. Therefore, in the absence of evidence to the contrary, aggressive medical therapy of PVD used in the general population should be adopted to the dialysis population. Thus, in all dialysis patients with PVD (including asymptomatic patients), smoking cessation, maintenance of LDL cholestrol < 100 mg/dl, maintenance of glycosylated HbA1c < 7.0 mg/dl in diabetics, control of blood pressure, use of ACE inhibitors and antiplatelet agents (aspirin or clopidogrel) should be considered [21].

There are several limitations to our study. First, even though the use of propensity scores might have reduced indication bias, residual bias might still exist. Therefore, all the analyses should be interpreted with caution. Second, clinical data such as the level and extent of arterial stenosis, extent of tissue necrosis, presence or absence of infection were not available. However, it might be reasonable to assume that patients with extensive tissue necrosis and severe infections underwent amputations rather than revascularization. Thus, it could still be inferred that a careful selection for revascularization of patients without severe disease might result in acceptable outcomes and save the limb. On the other hand, information on quality of life pre and post-procedures were also not available. It is possible that major amputation resulted in significant loss in quality of life and successful revascularization improved quality of life. Third, the presence or absence of gangrene was identified from ICD-9 CM codes. It is possible that the strong association of gangrene with amputation was not because gangrene was an indication for amputation rather patients whom underwent amputation were coded to have gangrene. We believe this is unlikely because 79% of all deaths within 30 days of procedure occurred in patients with an ICD-9-CM diagnosis of gangrene and it was also associated with 54% higher odds of death in the multivariable model. Fourth, ICD-9-CM codes on clinical indication were not present in 874 (22.2%) patients and thus this information is unavailable in those patients. However, as the death rate of these patients were not as high of those patients with gangrene, it is quite likely that the ICD-9-CM codes on clinical indication were not entered in patients without gangrene. Fifth, even though small, single center studies could obtain more information on primary graft patency and limb salvage, in the larger national sample of Medicare patients these data are unavailable. Thus, larger studies of national sample of Medicare patients and smaller single center studies complement each other. Finally, the limitations of this study include those of retrospective studies that use existent databases. Since only patients on Medicare defined the study population, it is possible that patients younger than 65 years who were not yet covered by Medicare were excluded introducing some selection bias. Further, the medical evidence form (form 2728) has been shown to have intermediate sensitivity but high specificity for comorbid conditions reported in the form [22].

## Conclusion

We conclude that amputation might carry significant morbidity and mortality in dialysis patients compared to revascularization procedures. More importantly, further studies are warranted to determine whether early diagnosis and aggressive medical therapy decrease the need for revascularization or amputation in dialysis patients with PVD.

## Competing interests

The author(s) declare that they have no competing interests.

## Authors' contributions

CL contributed to the conception and design of the study, interpreted the data and drafted the manuscript. NR contributed to the conception of the study and to preparation of the manuscript. LP participated in the design of the study and performed the statistical analysis. SB conceived the study, performed statistical analysis, interpreted the data and drafted the manuscript. All authors read and approved the final manuscript.

## Pre-publication history

The pre-publication history for this paper can be accessed here:



## Figures and Tables

**Table 1:** Baseline characteristics of the study population by procedure groups

	Amputated	Revascularized
N = 3942	N = 2046	N= 1896
**Demographics**		
Age in years, mean ± SD	69.4 ± 9.7	71.5 ± 8.1
Male Gender, n (%)	1148 (56)	1097 (58)
Race, n (%)		
Caucasians	1333 (65)	1464 (77)
African Americans	630 (31)	370(20)
Other	83 (4)	62 (3)
**ESRD**		
Diabetes as cause of ESRD, n (%)	1518 (74)	1011 (53)
Dialysis Modality, n (%)		
HD	1875 (92)	1777 (94)
PD	171 (8)	119 (6)
**Comorbidity**		
Coronary disease, n (%)	1077 (53)	893 (47)
Congestive heart failure, n (%)	1076 (53)	934 (49)
Cerebrovascular disease, n (%)	380 (19)	289 (15)
Smoker, n (%)	94 (5)	138 (7)
Cancer, n (%)	74 (4)	91 (5)
**Clinical Indications**		
Intermittent claudication	6 (0.3)	91 (4.8)
Resting pain	13 (0.6)	97 (5.1)
Ulcer	31 (1.5)	225 (11.9)
Gangrene	1850 (90.4)	755 (39.8)
Other/unknown	146 (7.1)	728 (38.4)
**Nutrition**		
BMI, mean ± SD	25.3 ± 5.6	24.7 ± 5.2
Serum Albumin, mean ± SD	3.0 ± 0.6	3.2 ± 0.6
**Functional Status**		
Require assistance to transfer, n (%)	139 (7)	30 (2)
Require assistance to ambulate, n (%)	342 (17)	113 (6)
**Other**		
Hematocrit, mean ± SD	27.8 ± 7.0	28.5 (6.8)

**Table 2:** Variables significantly associated with amputation in a multivariate logistic model

Covariate	Odds Ratio	95% confidence interval	P-value
5 year increase in age	0.90	0.86 – 0.94	<0.001
African American v Caucasian	1.52	1.27 – 1.81	<0.001
Diabetes as cause of ESRD	1.66	1.40 – 1.96	<0.001
Peritoneal dialysis	1.54	1.14 – 2.09	0.005
Gangrene v claudication	19.0	13.86 – 25.95	<0.001
Indication for amputation unknown/other v claudication	1.53	1.08 – 2.18	0.018
Each g/dl increase in serum albumin	0.73	0.64 – 0.83	<0.001
Needs assistance to transfer	1.72	1.00 – 2.94	0.049
Needs assistance to ambulate	2.59	1.89 – 3.53	<0.001

**Table 3:** 30-day and 1 year post-procedure mortality in amputation versus revascularization groups in the entire cohort and subgroups of likelihood of amputation

	30 day mortality	1 year mortality
	Odds ratio* (95%CI)	Odds ratio* (95%CI)
Entire cohort (n = 3942)		
Unadjusted	2.00 (1.64–2.44)	1.67 (1.48–1.90)
Adjusted**	1.85 (1.45–2.36)	1.46 (1.25–1.71)
Subgroup analyses by likelihood for amputation***		
Low likelihood group (n = 1314)	1.85 (1.06–3.23)	1.61 (1.14–2.26)
Moderate likelihood group (n = 1314)	2.05 (1.47–2.87)	1.55 (1.22–1.96)
High likelihood group (n = 1314)	1.56 (0.91–2.21)	1.23 (0.92–1.63)

*reference revascularization group

**adjusted for propensity scores, demographics (age, sex, race), dialysis modality, comorbid conditions (diabetes, coronary artery disease, congestive heart failure, cerebrovascular disease and malignancy), clinical indications (intermittent claudication, resting pain, ulcer, gangrene or other/unknown), hematocrit, nutritional status (BMI and serum albumin) and functional status (required assistance to transfer or ambulate).

***adjusted for all above variables except propensity score
